# Associations between air pollutants and acute exacerbation of drug-resistant tuberculosis: evidence from a prospective cohort study

**DOI:** 10.1186/s12879-024-09011-x

**Published:** 2024-01-23

**Authors:** Chan-Na Zhao, Zhiwei Xu, Peng Wang, Jie Liu, Rong Wang, Hai-Feng Pan, Fangjin Bao

**Affiliations:** 1 Department of Epidemiology and Biostatistics, School of Public Health, Anhui Medical University, 81 Meishan Road, 230032 Hefei, Anhui China; 2 School of Medicine and Dentistry, Griffith University, Gold Coast, Australia; 3 Teaching Center for Preventive Medicine, School of Public Health, Anhui Medical University, 81 Meishan Road, 230032 Hefei, Anhui China; 4 Department of Tuberculosis Control, Tuberculosis Control Institute of Anhui Province, 397 Jixi Road, 230022 Hefei, Anhui China

**Keywords:** Air pollutants, Drug-resistant tuberculosis, NO_2_

## Abstract

**Background:**

Short-term exposure to air pollution may trigger symptoms of drug-resistant tuberculosis (DR-TB) through stimulating lung tissue, damaging tracheobronchial mucosa, the key anti-mycobacterium T cell immune function, and production and release of inflammatory cytokines.

**Objective:**

To investigate the association between acute exacerbations of DR-TB and short-term residential exposure to air pollutants (PM_10_, PM_2.5_, SO_2_, NO_2_, CO and O_3_) based on a large prospective cohort in Anhui Province, China.

**Method:**

Patients were derived from a prospective cohort study of DR-TB in Anhui Province. All DR-TB patients underwent drug-susceptibility testing and prefecture-level reference laboratories confirmed their microbiologies. The case-crossover design was performed to evaluate the association between the risk of acute exacerbations of DR-TB and short-term residential exposure to air pollution.

**Results:**

Short-term NO_2_ exposure was significantly related to an elevated risk of first-time outpatient visit due to acute exacerbations of DR-TB(relative risk:1.159, 95% confidence interval:1.011 ~ 1.329). Stratification analyses revealed that the relationship between the risk of acute exacerbations and NO_2_ exposure was stronger in the elderly (age ≥ 65) DR-TB patients, and in individuals with a history of TB treatment.

**Conclusions:**

NO_2_ Exposure was significantly associated with an elevated risk of acute exacerbation of DR-TB in Anhui Province, China.

**Supplementary Information:**

The online version contains supplementary material available at 10.1186/s12879-024-09011-x.

## Introduction

Tuberculosis (TB) is the main cause of death from infectious diseases worldwide, causing 1.4 million deaths in 2021 [1]. Eight countries accounted for more than two thirds of global TB cases, and China ranked the 3rd among these eight countries [1]. Drug-resistant tuberculosis (DR-TB) is a kind of TB that has developed resistance to one or more anti-TB drugs. Treating DR-TB patients require toxic and expensive second-line drugs and 40% cases are treated unsuccessfully [1].

A growing number of researches have investigated connections between short-term air pollution exposure and TB risk [2–7]. For each 10-μg/m^3^ increase in SO_2_ with a 3-day lag, the risk of an initial TB outpatient visit was reduced by 2.0% [2]. Research also showed that the higher the concentration of PM_2.5_, PM_10_ and NO_2_, the higher the risk of tuberculosis [3–6]. Air pollutants could contribute to airway inflammation activation, immune imbalance and higher prevalence of respiratory symptoms including sore throat, cough, sputum, wheeze and dyspnea [8–10]. In clinical practice, the most visible sign of DR-TB aggravation is an escalation of respiratory symptoms like cough and sputum. Short-term exposure to air pollutants may trigger the symptoms of DR-TB by stimulating lung tissue, damaging tracheobronchial mucosa and the key anti-mycobacterium T cell immune function, and promoting the generation and secretion of inflammatory cytokines [11–13].

When analyzing the relationship between the acute exacerbation of DR-TB and the concentration of air pollutants, considering the lag effect of pollutants, the concentration of air pollutants in the period before the case was used. We conducted this case-crossover study, wishing to (1) assess the connection between the risk of acute exacerbation of DR-TB and short-term exposure to PM_10_, PM_2.5_, SO_2_, NO_2_, O_3_ and CO in Anhui Province, China; (2) investigate if there were any subpopulations particularly vulnerable to the impact of short-term air pollution exposure.

## Materials and methods

### Study design

A time-stratified case-crossover design was performed in this study. The design made each case act as a self-control. The case day referred to the date of first-time outpatient visits for acute exacerbations of DR-TB, and the control day was the case day’s same year, month, and day of week. For example, if the case day occurred on December 12, 2014 (Friday), then all other Fridays in December 2014 were selected as the control day.

### Study settings

**Fig. 1:**
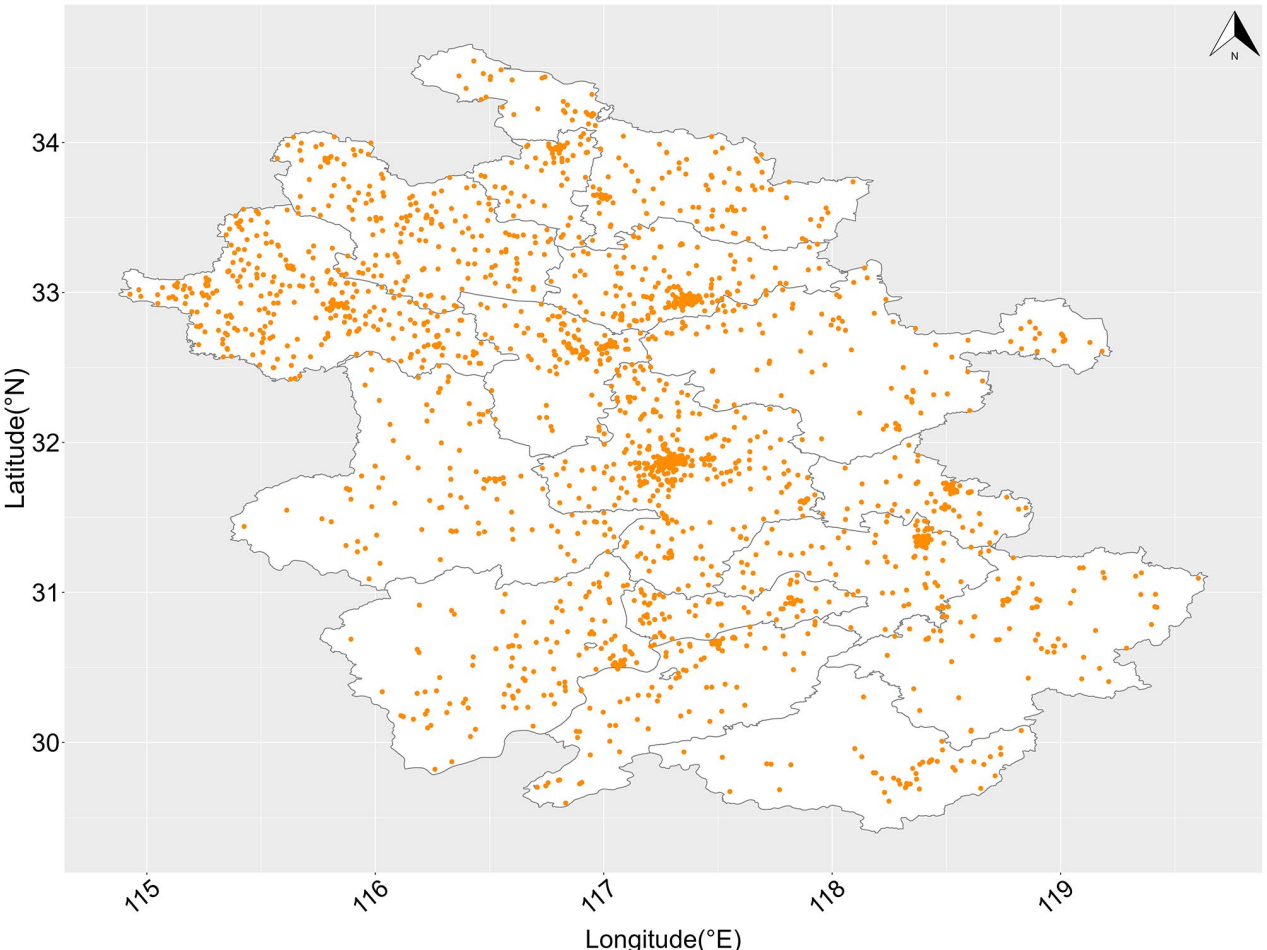
Spatial distributions of 2522 DR-TB cases’ residential addresses in Anhui province, China DR-TB, drug resistant tuberculosis

Anhui Province is located between 29°41′-34°38′N and 114°54′-119°37′E, covering an area of 140,100 km^2^ (Fig. 1). It is consisted of 16 cities and had a population of 61.13 million in 2021.

### Study population

The Anhui Province-Resistant Tuberculosis Cohort Study is a prospective cohort study conducted in Anhui, China. The cohort recruited all drug-resistant TB patients living in Anhui Province, China based on the China National Tuberculosis Information Management System (TBIMS). In accordance with China’s national TB control guidelines, any health facility that discovers a case or suspected case of TB must report it through TBIMS within 24 h. TBIMS records information of TB cases diagnosed based on mycobacterial culture, sputum smears and chest X-rays. The cohort was carried out from January 1, 2015. Participants from the start of the cohort to December 31, 2020 were enrolled in this study. The inclusion criteria for this study were as follows: first-time diagnosis of DR-TB, having symptoms of acute exacerbation, having drug-susceptibility testing (DST) results, completing interviews with demographic information, and reporting of a clear residential address. Of the patients who participated in the study, 39 did not provide a clear residential address or did not have laboratory records, so 2522 (94.7%) participants were included in the final analysis. Before the data collecting began, the Ethics Committee of Anhui Medical University gave its clearance.

### Drug susceptibility testing

All DR-TB cases included in this study had DST results. Prefecture-level reference laboratories provided microbiologic confirmation. DST was done using phenotypic susceptibility testing or Mycobacterium tuberculosis/rifampicin resistance test(GeneXpert MTB/RIF) at reference-level reference laboratories. Löwenstein-Jensen (LJ) slants were used in phenotypic DST. The indirect proportion method was used for phenotypic DST using a standardized and two 10-fold diluted dilutions of the inoculum with the anti-TB drug MIC tested. Drug resistance was defined when at least 1% of growth was observed at the drug MIC when compared to growth without drug. In the M.tb genome, GeneXpert MTB/RIF identified particular DNA alterations linked to resistance to particular anti-TB medications. The Xpert MTB/RIF Ultra or Xpert XDR (Cepheid, Sunnyvale, CA, USA), or the Abbott RealTime MTB and RealTime MTB RIF/INH (by Abbott, Abbott Park, Illinois, USA) were utilized in the GeneXpert MTB/RIF test.

### Air pollutants data

The China High Air Pollutants (CHAP) grid dataset was used to determine the average concentrations of air pollutants in Anhui Province from 2015 to 2020 [14–16]. Specifically, in consideration of the spatial and temporal heterogeneity of air pollution, average concentrations of air pollution with a 1 km*1km spatial resolution were estimated using artificial intelligence. For PM_10_, PM_2.5_, SO_2_, CO, NO_2_, and O_3,_ the cross-validation coefficient of determination was 0.90, 0.92, 0.84, 0.80, 0.84 and 0.87 respectively. Each DR-TB case’s daily exposure on case days and control days was assessed based on the level of air pollution at their residential address.

The 24-hour relative humidity (RH, %) and the mean temperature (^◦^C) were obtained from ERA5 data. The ERA5 dataset combines a global climate model with situ and satellite observations [17]. We matched the temperature and RH for each case according to their residential address.

### Definitions

First-time diagnosis was defined as the diagnosis of DR-TB through laboratory testing at the first-time outpatient visit. High-risk DR-TB cases were those who were registered in the TBIMS as having initial treatment failure, retreatment failure, relapse and return, chronic and smear-positive at the end of the second or third month (hereinafter called as ‘high-risk DR-TB subgroup’). It should be noted that high-risk DR-TB cases were classified according to the patient’s previous treatment history of TB. History of TB treatment referred to the treatment history of TB other than DR-TB.

### Statistical analysis

To quantify the relationship between first-time outpatient visit for DR-TB and air pollutants in Anhui Province of China, the study performed the time-stratified case-crossover design [18]. Each case acted as his/ her own control. The lagged non-linear exposure–response patterns were captured by a distributed lag nonlinear model (DLNM) with conditional Poisson regression [19].

Our preliminary data analysis showed that the relationship between the risk of acute exacerbations of DR-TB and air pollution exposure lasted for about one week, so we used a maximum lag of 7 days in the main analyses. Daily relative humidity, average temperature and holidays were controlled as potential confounders. To determine potentially vulnerable populations, we carried out stratified analyses by sex, occupation, age (< 65 years old, ≥ 65 years old), high-risk DR-TB (vs. low-risk DR-TB), history of treatment and season (cold season: October-March, and warm season: April-September) to evaluate their potential effects. To test the models’ robustness, we conducted two-pollutant models. All analyses were performed using R version 4.2.1.

## Results

### Summary statistics of the DR-TB cases

From 2015 to 2020, there were 2,522 newly diagnosed DR-TB cases, including 1,935 males (76.7%) and 587 females (23.3%). The average age of DR-TB cases was 50.77 ± 17.14 years. Among these cases, 23.8% were ≥ 65 years and 76.2% were < 65 years (Table 1). Spatial distributions of 2522 DR-TB cases’ residential addresses were shown in Fig. 1.

**Table 1:** Basic characteristics of the study population, 2015 to 2020

Data	N(%)
Total	2522(100)
Rifampin resistance	2507(99.4)
Isoniazid resistance	1868(74.1)
Ethambutol resistance	586(23.2)
Gender	
Female	587(23.3)
Male	1935(76.7)
Age (years)	
>=65	600(23.8)
< 65	1922(76.2)
High-risk group	1224(48.5)
Low-risk group	1298 (51.5)
History of TB treatment	
Yes	2262 (89.7)
No	260 (10.3)
Season	
Warm	1376(54.6)
Cold	1146 (45.4)
Occupation	
Farmer	1597(63.2)
Others	927 (36.8)

### Statistics of meteorologic factors and air pollutants

Summary statistics of meteorologic conditions and air pollutants from 2015 to 2020 in Anhui Province were shown in Table 2. The mean concentrations of PM_10,_ PM_2.5_, SO_2_, NO_2_, CO, and O_3_ were 54.5 μg/m^3^,36.3 μg/m^3^, 11.6 μg/m^3^, 17.4 μg/m^3^, 0.4 mg/m^3^, and 54.1 μg/m^3^, respectively.

**Table 2:** Distribution of air pollutants and meteorologic conditions during case and control days in Anhui Province, China, 2015–2020

Variable	Means	SD	Min	P25	Median	P75	Max	IQR
Air pollutants								
PM25(μ/m^3^)	55	31.6	1	32.6	47.8	68.9	410.3	36.3
PM10(μ/m^3^)	88.9	44.5	6	56.8	80.7	111.3	797.3	54.5
SO2(μ/m^3^)	18.8	11	1.4	11.4	16.4	23	621.4	11.6
NO2(μ/m^3^)	33.5	14.7	1.2	23.2	30.1	40.6	280.3	17.4
O3(μ/m^3^)	92.2	39.9	2.1	62.5	84	116.7	467.6	54.1
CO(mg/m^3^)	0.9	0.3	0	0.7	0.8	1.1	10.8	0.4
Meteorologic condition								
Temperature(℃)	16.6	9.2	-11.1	8.5	17.6	24.5	35.1	16
Relative Humidity (%)	73.1	13.9	20.7	63.6	74.3	83.6	100	20

Definition of abbreviations: CO = carbon monoxide; IQR = interquartile range; Max = maximum; Min = minimum; NO2 = nitrogen dioxide; O3 = ozone; P25 = the 25th percentile; P75 = the 75th percentile; PM2.5 = particulate matter with an aerodynamic diameter < 2.5 mm; PM10 = particulate matter with an aerodynamic diameter < 10 mm; SO2 = sulfur dioxide

### Association between the risks of acute exacerbations of DR-TB and air pollutants

**Fig. 2:**
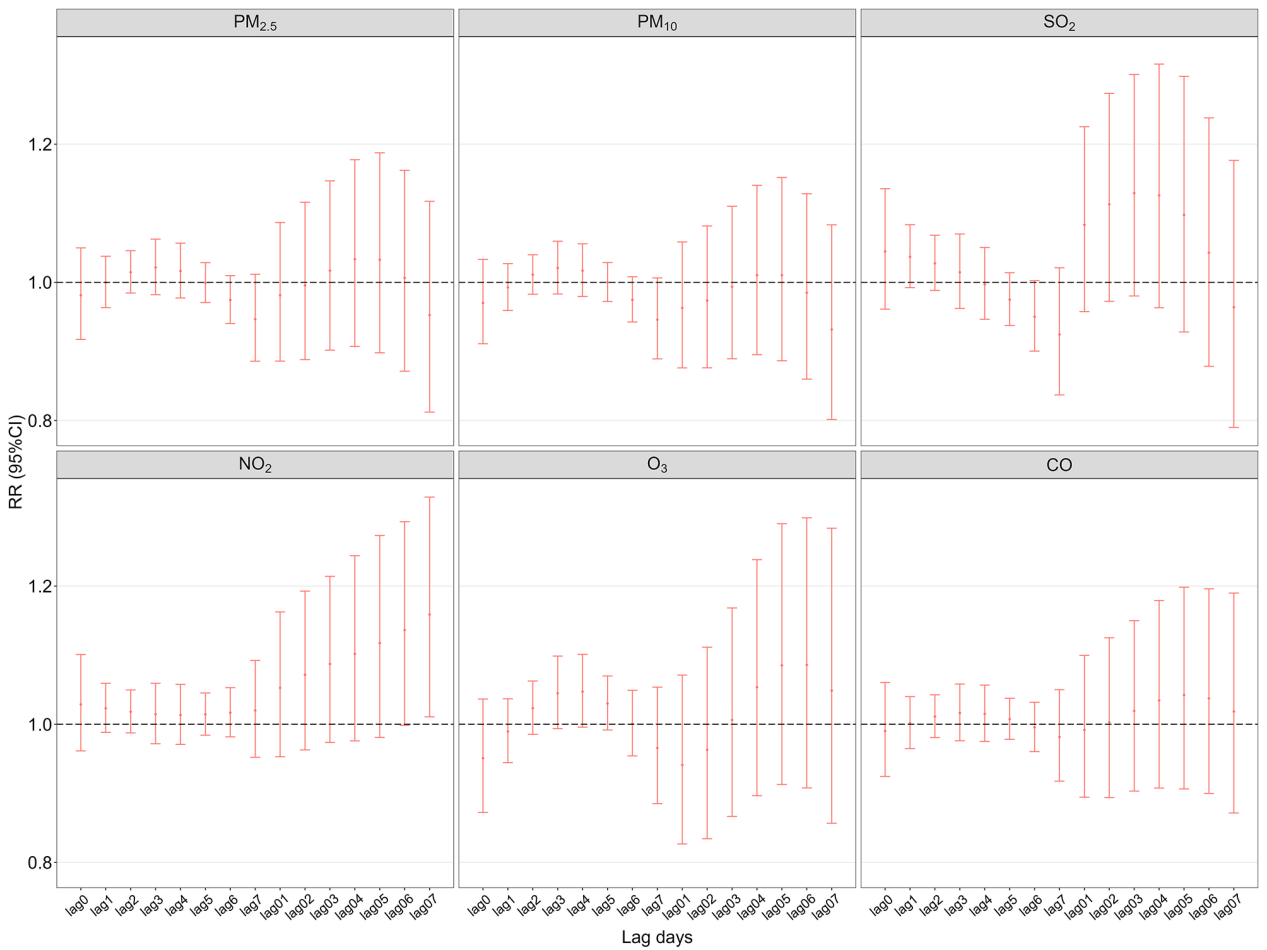
RR (95% CIs) for the association between first-time outpatient visits due to acute exacerbation of DR-TB and air pollutants concentrations with each IQR increase based on single-pollutant models DR-TB, drug resistant tuberculosis; RR, Relative risks; CIs, confidence intervals

NO_2_ exposure was related to an increased risk of initial outpatient visits due to acute exacerbation of DR-TB (RR = 1.159, 95%CI:1.011 ~ 1.329, lag 07). No significant association was discovered between acute exacerbation of DR-TB and exposure to the other five air pollutants. (Fig. 2, Table S1). The RRs and 95% CIs of initial outpatient visits for acute DR-TB exacerbation associated with each IQR increase of exposures to air pollutants were presented in Table 3.

**Table 3:** Relative risk(RR) and 95% confidence interval (CI) for the association between acute exacerbation of DR-TB and air pollutants concentrations with each IQR increase in different subgroups at lag07 day

	PM_2.5_	PM_10_	SO_2_	NO_2_	O_3_	CO
Total	0.953 (0.812–1.117)	0.932 (0.801–1.083)	0.964 (0.790–1.176)	1.159 (1.011–1.329)*	1.049 (0.857–1.284)	1.018 (0.872–1.190)
Male	0.949 (0.792–1.138)	0.927 (0.780–1.102)	0.901 (0.716–1.134)	1.180 (1.009–1.380)*	0.968 (0.766–1.222)	0.976 (0.816–1.166)
Female	0.977 (0.699–1.365)	0.983 (0.914–1.057)	1.189 (0.791–1.789)	1.064 (0.812–1.395)	1.384 (0.917–2.088)	1.185 (0.861–1.632)
*P*_difference_	0.883	0.542	0.246	0.516	0.138	0.298
< 65 years old	0.919 (0.764–1.104)	0.927 (0.779–1.103)	0.869 (0.691–1.093)	1.083 (0.926–1.268)	0.911 (0.721–1.151)	0.973 (0.813–1.165)
≥ 65 years old	1.041 (0.752–1.441)	0.985 (0.783–1.238)	1.456 (0.953–2.223)	1.395 (1.048–1.856)*	1.609 (1.063–2.436)*	1.146 (0.836–1.572)
*P*_difference_	0.511	0.680	**0.036**	0.129	**0.019**	0.377
Farmers	0.979 (0.800-1.198)	0.894 (0.738–1.083)	0.882 (0.675–1.154)	1.159 (0.975–1.378)	1.102 (0.855–1.419)	0.989 (0.810–1.207)
Others	0.911 (0.702–1.184)	0.996 (0.849–1.168)	1.096 (0.806–1.489)	1.164 (0.929–1.459)	0.935 (0.662–1.320)	1.072 (0.834–1.378)
*P*_difference_	0.672	0.396	0.298	0.975	0.452	0.621
With high-risk	1.086 (0.865–1.363)	0.983 (0.793–1.218)	0.990 (0.739–1.326)	1.219 (1.005–1.480)*	1.156 (0.860–1.554)	1.065 (0.856–1.325)
With low-risk	0.832 (0.662–1.046)	0.878 (0.708–1.089)	0.950 (0.717–1.257)	1.106 (0.910–1.345)	0.982 (0.743–1.299)	0.969 (0.774–1.213)
*P*_difference_	0.105	0.465	0.843	0.488	0.434	0.554
With a history of treatment	0.941 (0.796–1.113)	0.941 (0.801–1.105)	0.973 (0.789–1.198)	1.201 (1.041–1.386)*	1.037 (0.838–1.284)	1.001 (0.851–1.178)
Without history of treatment	1.205 (0.701–2.072)	0.948 (0.605–1.487)	0.856 (0.432–1.694)	0.802 (0.491–1.308)	1.097 (0.561–2.145)	1.381 (0.782–2.440)
*P*_difference_	0.393	0.975	0.726	0.120	0.876	0.286
Warm	0.899 (0.631–1.282)	0.743 (0.559–0.987)*	0.939 (0.697–1.264)	1.132 (0.878–1.459)	1.272 (0.973–1.663)	1.100 (0.837–1.446)
Cold	0.924 (0.768–1.113)	0.977 (0.812–1.176)	0.958 (0.725–1.264)	1.134 (0.957–1.343)	0.810 (0.572–1.147)	0.962 (0.792–1.170)
*P*_difference_	0.893	0.113	0.924	0.990	**0.044**	0.436

Definition of abbreviations: CO = carbon monoxide; DR-TB = Drug resistant tuberculosis; IQR = interquartile range; NO_2_ = nitrogen dioxide; O_3_ = ozone; PM_2.5_ = particulate matter with an aerodynamic diameter < 2.5 mm; PM_10_ = particulate matter with an aerodynamic diameter < 10 mm; SO_2_ = sulfur dioxide

This model was adjusted for daily relative humidity, average temperature, and holidays. *:*P* < 0.05

### Stratified analyses

Stratified analyses were shown in Figure S1-S6. The associations between the risk of acute exacerbation of DR-TB and air pollutants exposure did not differ by sex (Table 3, Figure S1) or occupation (Table 3, Figure S3). However, the effect of SO_2_ exposure appeared to be stronger in those ≥ 65 years (vs. those < 65 years) (Table 3, Figure S2), and in high-risk DR-TB cases (vs. low-risk DR-TB cases) (Table 3, Figure S4). Moreover, the effect of NO_2_ exposure appeared to be stronger in the elderly (vs. those < 65 years), and in individuals with a history of TB treatment (vs. those without history of treatment) (Table 3, Figure S2, Figure S5). The effect of O_3_ exposure was stronger in those DR-TB patients who first visited clinics in warm season (vs. cold season) (Table 3, Figure S6).

### Sensitivity analysis

The sensitivity analysis revealed that the primary results in the two-pollutant models remained robust(Tables S2-S3).

## Discussion

This study has observed three intriguing findings. First, short-term exposure to NO_2_ was significantly related to an increased risk of acute exacerbation of DR-TB. Second, two subpopulations (those ≥ 65 years and high-risk DR-TB cases) appeared more vulnerable to the effect of SO_2_. Third, DR-TB patients who first visited clinics in the warm season were more vulnerable to the effect of O_3_.

To the best of our knowledge, this is the first study that examines the association between exacerbation of DR-TB symptoms and short-term air pollution exposure. A rising number of investigations have investigated the relationships between air pollutants and TB. Positive associations were found between exposure to PM_2.5_ [5, 20–23], PM_10_ [6, 21–24], NO_2_ [3–5, 22, 25], SO_2_ [3, 21–23, 26, 27], CO [22, 23] or O_3_ [23] and the risk of TB. However, some studies also found a negative association between exposure to O_3_ [22, 25], SO_2_ [2, 5] and PM_10_ [28] and the risk of TB. A cohort study also showed a significant positive relationship between O_3_ exposure and the incidence of DR-TB [29]. Another study found that among patients with multidrug-resistant TB, greenness reduced air pollution-related mortality [30]. The two studies above suggested that environmental factors may determine the outcome of DR-TB patients. Several studies reported that NO_2_ inhalation can trigger proinflammatory responses and promote pulmonary infections, and impair host defence against infection in the lung [31, 32]. It is suggested that NO_2_ exposure may play a role in lung injury by inducing the infiltration of alveolar macroscopic (AM) subgroups [33]. The infiltration of AM subgroups produces a large amount of IL-10 to play an anti-inflammatory role and selectively express some matrix metalloproteinases, which may contribute to the pathophysiology of NO_2_-induced lung damage [33].

The underlying mechanisms of increased risk of DR-TB owing to air pollution exposure are multi-factorial. Firstly, the immune system consists of a variety of dedicated immune cells. Air pollutants can act on these immune cells, such as epithelial cells [34] and parenchymal cells [35], dendritic cells [36] and lymphocytes [37], and granulocytes [38, 39]. Additionally, air pollutants cause oxidative stress [40] and activate a cascade of immune dysfunction and pro-inflammatory pathways [41]. Pollutants can stimulate cells through polycyclic aromatic hydrocarbons (PAH) sensing pathway [42], Toll like receptors (TLRs) [43] and oxidative and nitrosative stress pathways [44], which in turn stimulate pro-inflammatory intracellular signaling pathways like MAPK and NF-kB pathways.

In the current study, female cases were especially vulnerable to SO2 and O3, while the male cases were especially vulnerable to NO_2_. This result shows that the impact of air pollutants on DR-TB may vary by gender. On stratification by age, high PM_2.5_ and SO_2_ exposure were inversely associated with acute exacerbations of DR-TB for young patients. For old patients, high SO2, NO2 and O3 exposure were positively associated with acute exacerbations of DR-TB. This may be because the elderly may be weaker and have basic diseases, leading to greater vulnerability to air pollutants. The current study revealed that high-risk subgroup was especially vulnerable to PM_2.5_, SO_2_ and NO_2_. This may because these people’s lungs have been damaged, and they are more sensitive to air pollutants than others. In addition, we found that SO_2_ has opposite effects on subgroup patients. The positive effects of SO_2_ were observed at lag1, lag2 and lag01-lag03 days among old patients, but negative effects were observed at lag6 and lag7 among young patients. Among the patients without a history of treatment, the positive effects of SO_2_ were observed at lag0 and lag01, but the negative effects of SO_2_ were observed at lag3-5. In the group of high-risk population, the positive effects of SO_2_ were observed at lag1, lag2 and lag01-lag04 days, but negative effects were observed at lag6. After careful observation, we found that short-exposure to SO_2_ may increase the risk of DR-TB, while long-exposure to SO_2_ may reduce acute exacerbations of DR-TB. This may be explained by the reason that, to some extent, SO_2_ can inhibit the activity of mycobacterium TB complex [45]. Moreover, in the warm seasons, high O_3_ expose increased the risk of acute exacerbations of DR-TB, which may due to higher O_3_ concentration in warm seasons. If our findings are confirmed in other regions, it will bring obvious clinical and public health significance, because we can prevent DR-TB by controlling the concentration of air pollutants.

The WHO 2021 global air quality guideline values for SO_2_, NO_2_ and O_3_ are 40 μg/m^3^, 25 μg/m^3^ and 100 μg/m^3^ respectively, while the level 1 limits in China (GB 3095 − 2012) are 50 μg/m^3^, 80 μg/m^3^ and 100 μg/m^3^ respectively. Although the average concentrations of pollutants in this study were lower than WHO and China standards, we still observed a risk to DR-TB from air pollutants. Therefore, we should boost industrial structure optimisation and adjustment, and withdraw the backward production capacity of key industries in accordance with the law; promote the transformation of green and low-carbon energy, strictly control the growth of coal consumption, and vigorously develop new and clean energy; carry out the upgrading of traditional industrial clusters.

This study has some strengths. First, we provided estimations of the impacts of six pollutants on DR-TB and conducted subgroup analyses. Second, high-spatial-resolution air pollution data were used in this investigation to estimate each patient’s exposure. Most early studies used urban average air pollution concentrations for exposure assessment. Third, this study is a provincial level research with a large sample size, including 2,522 newly diagnosed DR-TB cases. Fourth, the data was derived from the DR-TB cohort study in Anhui Province based on TBIMS. Based on China’s national TB control guidelines, any health establishment that discovers TB cases or probable cases must report them via TBIMS. Therefore, through this system, we can accurately grasp the incidence of DR-TB in Anhui Province.

This study also has some limitations. First, the incidence of DR-TB reported in our study may be underestimated. This low detection rate is an essential challenge in the therapy and prevention of DR-TB. The WHO report showed that there were about 450 000 incident patients of multi-drug resistance/rifampin resistance TB in 2021 worldwide, while only 162,000 were diagnosed [1]. Second, the study used outdoor air pollution exposure to assess the impact, but patients may spend most of their time indoors. So, exposure measurement bias cannot be excluded. Third, considering that a decrease in sample size may lead to model instability and unreliable results, we did not conduct subgroup analysis on subgroups with smaller sample sizes after grouping, such as subgroups with worsening respiratory symptoms after treatment, more refined occupational groups, and different types of drug resistance.

## Conclusion

NO_2_ exposure is associated with an elevated risk of exacerbation of DR-TB. Elderly DR-TB patients, high-risk DR-TB cases and patients with a history of TB treatment need to be made aware of the risk posed by air pollution exposure.

### Electronic supplementary material

Below is the link to the electronic supplementary material.

**Supplementary Material 1: Table S1** RR (95% CIs) for the association between first-time outpatient visits for acute exacerbations of DR-TB and air pollutants concentrations with each IQR increase based on single-pollutant models. **Table S2** Single-lag RR (95% CIs) for the association between first-time outpatient visits for acute exacerbations of DR-TB and air pollutants concentrations with each IQR increase based on two-pollutants models. **Table S3** Cumulative RR (95% CIs) for the association between first-time outpatient visits for acute exacerbations of DR-TB and air pollutants concentrations with each IQR increase based on two-pollutants models. **Figure S1**. RR (95% CIs) for the association between first-time outpatient visits for acute exacerbations of DR-TB and air pollutants concentrations with each IQR increase based on single-pollutant models stratified by gender. **Figure S2**. RR (95% CIs) for the association between first-time outpatient visits for acute exacerbations of DR-TB and air pollutants concentrations with each IQR increase based on single-pollutant models stratified by age. **Figure S3**. RR (95% CIs) for the association between first-time outpatient visits for acute exacerbations of DR-TB and air pollutants concentrations with each IQR increase based on single-pollutant models stratified by occupation. **Figure S4**. RR (95% CIs) for the association between first-time outpatient visits for DR-TB and air pollutants concentrations with each IQR increase based on single-pollutant models stratified by high-risk subgroup. **Figure S5**. RR (95% CIs) for the association between first-time outpatient visits for DR-TB and air pollutants concentrations with each IQR increase based on single-pollutant models stratified by history of treatment. **Figure S6**. RR (95% CIs) for the association between first-time outpatient visits for DR-TB and air pollutants concentrations with each IQR increase based on single-pollutant models stratified by season

## Data Availability

The datasets used and/or analysed during the current study available from the corresponding author on reasonable request.
