# Supplementary material for: Associations between air pollutants and acute exacerbation of drug-resistant tuberculosis: evidence from a prospective cohort study
Source: BMC Infect Dis. 2024 Jan 23;24:121. doi: 10.1186/s12879-024-09011-x (PMC10807089; doi:10.1186/s12879-024-09011-x)
Supplement: Supplementary file 1 — Supplementary Material 1: Table S1 RR (95% CIs) for the association between first-time outpatient visits for acute exacerbations of DR-TB and air pollutants concentrations with each IQR increase based on single-pollutant models. Table S2 Single-lag RR (95% CIs) for the association between first-time outpatient visits for acute exacerbations of DR-TB and air pollutants concentrations with each IQR increase based on two-pollutants models. Table S3 Cumulative RR (95% CIs) for the association between first-time outpatient visits for acute exacerbations of DR-TB and air pollutants concentrations with each IQR increase based on two-pollutants models. Figure S1. RR (95% CIs) for the association between first-time outpatient visits for acute exacerbations of DR-TB and air pollutants concentrations with each IQR increase based on single-pollutant models stratified by gender. Figure S2. RR (95% CIs) for the association between first-time outpatient visits for acute exacerbations of DR-TB and air pollutants concentrations with each IQR increase based on single-pollutant models stratified by age. Figure S3. RR (95% CIs) for the association between first-time outpatient visits for acute exacerbations of DR-TB and air pollutants concentrations with each IQR increase based on single-pollutant models stratified by occupation. Figure S4. RR (95% CIs) for the association between first-time outpatient visits for DR-TB and air pollutants concentrations with each IQR increase based on single-pollutant models stratified by high-risk subgroup. Figure S5. RR (95% CIs) for the association between first-time outpatient visits for DR-TB and air pollutants concentrations with each IQR increase based on single-pollutant models stratified by history of treatment. Figure S6. RR (95% CIs) for the association between first-time outpatient visits for DR-TB and air pollutants concentrations with each IQR increase based on single-pollutant models stratified by season [file 12879_2024_9011_MOESM1_ESM.docx]

Table S1 RR (95% CIs) for the association between first-time outpatient visits for acute exacerbations of DR-TB and air pollutants concentrations with each IQR increase based on single-pollutant models

| Air pollutants | Single day | RR(95%CI) | Multi-day | RR(95%CI) |
| --- | --- | --- | --- | --- |
| PM_2.5_ |  |  |  |  |
|  | 0 | 0.981 (0.917-1.050) | - | - |
|  | 1 | 1.000 (0.963-1.038) | 01 | 0.981 (0.886-1.087) |
|  | 2 | 1.015 (0.985-1.046) | 02 | 0.996 (0.888-1.116) |
|  | 3 | 1.021 (0.982-1.063) | 03 | 1.017 (0.902-1.147) |
|  | 4 | 1.016 (0.977-1.057) | 04 | 1.033 (0.907-1.177) |
|  | 5 | 0.999 (0.971-1.028) | 05 | 1.033 (0.898-1.187) |
|  | 6 | 0.974 (0.940-1.010) | 06 | 1.006 (0.871-1.162) |
|  | 7 | 0.947 (0.886-1.012) | 07 | 0.953 (0.812-1.117) |
| PM_10_ |  |  |  |  |
|  | 0 | 0.970 (0.911-1.033) | - | - |
|  | 1 | 0.993 (0.959-1.027) | 01 | 0.963 (0.876-1.058) |
|  | 2 | 1.011 (0.983-1.040) | 02 | 0.974 (0.876-1.082) |
|  | 3 | 1.021 (0.983-1.060) | 03 | 0.994 (0.889-1.110) |
|  | 4 | 1.017 (0.980-1.056) | 04 | 1.010 (0.895-1.140) |
|  | 5 | 1.000 (0.972-1.028) | 05 | 1.010 (0.886-1.152) |
|  | 6 | 0.975 (0.943-1.008) | 06 | 0.985 (0.860-1.128) |
|  | 7 | 0.946 (0.889-1.006) | 07 | 0.932 (0.801-1.083) |
| SO_2_ |  |  |  |  |
|  | 0 | 1.045 (0.961-1.136) | - | - |
|  | 1 | 1.037 (0.992-1.083) | 01 | 1.083 (0.958-1.225) |
|  | 2 | 1.027 (0.988-1.068) | 02 | 1.113 (0.973-1.273) |
|  | 3 | 1.015 (0.962-1.070) | 03 | 1.129 (0.980-1.301) |
|  | 4 | 0.997 (0.946-1.050) | 04 | 1.126 (0.963-1.316) |
|  | 5 | 0.975 (0.938-1.014) | 05 | 1.098 (0.928-1.298) |
|  | 6 | 0.950 (0.900-1.003) | 06 | 1.043 (0.878-1.238) |
|  | 7 | 0.924 (0.837-1.021) | 07 | 0.964 (0.790-1.176) |
| NO_2_ |  |  |  |  |
|  | 0 | 1.029 (0.961-1.101) | - | - |
|  | 1 | 1.023 (0.988-1.059) | 01 | 1.053 (0.953-1.163) |
|  | 2 | 1.018 (0.987-1.050) | 02 | 1.072 (0.963-1.193) |
|  | 3 | 1.015 (0.972-1.059) | 03 | 1.087 (0.974-1.214) |
|  | 4 | 1.013 (0.971-1.058) | 04 | 1.102 (0.976-1.244) |
|  | 5 | 1.014 (0.984-1.045) | 05 | 1.118 (0.981-1.273) |
|  | 6 | 1.017 (0.982-1.053) | 06 | 1.136 (0.998-1.293) |
|  | 7 | 1.020 (0.952-1.092) | 07 | **1.159 (1.011-1.329)** |
| O_3_ |  |  |  |  |
|  | 0 | 0.951 (0.872-1.037) | - | - |
|  | 1 | 0.990 (0.944-1.037) | 01 | 0.941 (0.827-1.071) |
|  | 2 | 1.023 (0.985-1.063) | 02 | 0.963 (0.834-1.112) |
|  | 3 | 1.045 (0.994-1.099) | 03 | 1.006 (0.866-1.168) |
|  | 4 | 1.047 (0.996-1.101) | 04 | 1.054 (0.897-1.238) |
|  | 5 | 1.030 (0.992-1.070) | 05 | 1.085 (0.913-1.290) |
|  | 6 | 1.001 (0.954-1.049) | 06 | 1.086 (0.908-1.299) |
|  | 7 | 0.966 (0.885-1.054) | 07 | 1.049 (0.857-1.284) |
| CO |  |  |  |  |
|  | 0 | 0.990 (0.925-1.060) | - | - |
|  | 1 | 1.002 (0.965-1.040) | 01 | 0.992 (0.894-1.100) |
|  | 2 | 1.011 (0.981-1.043) | 02 | 1.003 (0.894-1.125) |
|  | 3 | 1.016 (0.976-1.058) | 03 | 1.019 (0.903-1.150) |
|  | 4 | 1.015 (0.975-1.057) | 04 | 1.035 (0.908-1.179) |
|  | 5 | 1.007 (0.978-1.038) | 05 | 1.042 (0.906-1.198) |
|  | 6 | 0.995 (0.960-1.032) | 06 | 1.037 (0.900-1.196) |
|  | 7 | 0.982 (0.918-1.050) | 07 | 1.018 (0.872-1.190) |

Definition of abbreviations: CO, carbon monoxide; CIs, confidence intervals; DR-TB, Drug resistant tuberculosis; IQR, interquartile range; NO_2_, nitrogen dioxide; O_3_,ozone; PM_2.5_, particulate matter with an aerodynamic diameter <2.5 mm; PM_10_, particulate matter with an aerodynamic diameter <10 mm; RR, Relative risks; SO_2_, sulfur dioxide.

This model was adjusted for daily relative humidity, average temperature, and holidays.

Table S2 Single-lag RR (95% CIs) for the association between first-time outpatient visits for acute exacerbations of DR-TB and air pollutants concentrations with each IQR increase based on two-pollutants models.

| Air pollutants | Adjusted pollutants | Lag | RR(95%CI) |
| --- | --- | --- | --- |
| PM_2.5_ | PM_10_ | 0 | 1.034 (0.903-1.184) |
| PM_2.5_ | PM_10_ | 1 | 1.025 (0.952-1.105) |
| PM_2.5_ | PM_10_ | 2 | 1.018 (0.955-1.084) |
| PM_2.5_ | PM_10_ | 3 | 1.010 (0.929-1.099) |
| PM_2.5_ | PM_10_ | 4 | 1.004 (0.924-1.091) |
| PM_2.5_ | PM_10_ | 5 | 0.999 (0.939-1.062) |
| PM_2.5_ | PM_10_ | 6 | 0.994 (0.925-1.069) |
| PM_2.5_ | PM_10_ | 7 | 0.990 (0.865-1.133) |
| PM_2.5_ | NO_2_ | 0 | 0.964 (0.896-1.036) |
| PM_2.5_ | NO_2_ | 1 | 0.987 (0.947-1.027) |
| PM_2.5_ | NO_2_ | 2 | 1.005 (0.972-1.039) |
| PM_2.5_ | NO_2_ | 3 | 1.014 (0.971-1.059) |
| PM_2.5_ | NO_2_ | 4 | 1.009 (0.967-1.053) |
| PM_2.5_ | NO_2_ | 5 | 0.989 (0.958-1.022) |
| PM_2.5_ | NO_2_ | 6 | 0.960 (0.923-1.000) |
| PM_2.5_ | NO_2_ | 7 | 0.928 (0.863-0.999) |
| PM_2.5_ | SO_2_ | 0 | 0.972 (0.907-1.042) |
| PM_2.5_ | SO_2_ | 1 | 0.994 (0.957-1.033) |
| PM_2.5_ | SO_2_ | 2 | 1.012 (0.981-1.044) |
| PM_2.5_ | SO_2_ | 3 | 1.022 (0.982-1.064) |
| PM_2.5_ | SO_2_ | 4 | 1.019 (0.980-1.061) |
| PM_2.5_ | SO_2_ | 5 | 1.005 (0.975-1.035) |
| PM_2.5_ | SO_2_ | 6 | 0.982 (0.946-1.019) |
| PM_2.5_ | SO_2_ | 7 | 0.956 (0.893-1.023) |
| PM_2.5_ | O_3_ | 0 | 0.988 (0.922-1.059) |
| PM_2.5_ | O_3_ | 1 | 1.001 (0.963-1.040) |
| PM_2.5_ | O_3_ | 2 | 1.010 (0.979-1.042) |
| PM_2.5_ | O_3_ | 3 | 1.014 (0.973-1.056) |
| PM_2.5_ | O_3_ | 4 | 1.008 (0.968-1.049) |
| PM_2.5_ | O_3_ | 5 | 0.993 (0.963-1.023) |
| PM_2.5_ | O_3_ | 6 | 0.972 (0.937-1.008) |
| PM_2.5_ | O_3_ | 7 | 0.949 (0.887-1.016) |
| PM_2.5_ | CO | 0 | 0.980 (0.895-1.074) |
| PM_2.5_ | CO | 1 | 1.000 (0.951-1.051) |
| PM_2.5_ | CO | 2 | 1.015 (0.974-1.057) |
| PM_2.5_ | CO | 3 | 1.020 (0.967-1.076) |
| PM_2.5_ | CO | 4 | 1.012 (0.960-1.067) |
| PM_2.5_ | CO | 5 | 0.989 (0.951-1.028) |
| PM_2.5_ | CO | 6 | 0.958 (0.912-1.005) |
| PM_2.5_ | CO | 7 | 0.923 (0.843-1.011) |
| PM_10_ | PM_2.5_ | 0 | 0.945 (0.833-1.073) |
| PM_10_ | PM_2.5_ | 1 | 0.973 (0.908-1.042) |
| PM_10_ | PM_2.5_ | 2 | 0.997 (0.939-1.058) |
| PM_10_ | PM_2.5_ | 3 | 1.012 (0.935-1.096) |
| PM_10_ | PM_2.5_ | 4 | 1.013 (0.936-1.098) |
| PM_10_ | PM_2.5_ | 5 | 1.001 (0.943-1.063) |
| PM_10_ | PM_2.5_ | 6 | 0.980 (0.915-1.049) |
| PM_10_ | PM_2.5_ | 7 | 0.954 (0.842-1.081) |
| PM_10_ | NO_2_ | 0 | 0.949 (0.886-1.016) |
| PM_10_ | NO_2_ | 1 | 0.977 (0.941-1.015) |
| PM_10_ | NO_2_ | 2 | 1.001 (0.970-1.033) |
| PM_10_ | NO_2_ | 3 | 1.014 (0.973-1.056) |
| PM_10_ | NO_2_ | 4 | 1.010 (0.970-1.053) |
| PM_10_ | NO_2_ | 5 | 0.991 (0.960-1.022) |
| PM_10_ | NO_2_ | 6 | 0.961 (0.925-0.998) |
| PM_10_ | NO_2_ | 7 | 0.926 (0.865-0.992) |
| PM_10_ | SO_2_ | 0 | 0.963 (0.903-1.027) |
| PM_10_ | SO_2_ | 1 | 0.988 (0.954-1.023) |
| PM_10_ | SO_2_ | 2 | 1.009 (0.980-1.038) |
| PM_10_ | SO_2_ | 3 | 1.021 (0.983-1.061) |
| PM_10_ | SO_2_ | 4 | 1.019 (0.981-1.059) |
| PM_10_ | SO_2_ | 5 | 1.004 (0.976-1.033) |
| PM_10_ | SO_2_ | 6 | 0.980 (0.948-1.015) |
| PM_10_ | SO_2_ | 7 | 0.953 (0.895-1.015) |
| PM_10_ | O_3_ | 0 | 0.976 (0.915-1.040) |
| PM_10_ | O_3_ | 1 | 0.994 (0.960-1.029) |
| PM_10_ | O_3_ | 2 | 1.008 (0.979-1.038) |
| PM_10_ | O_3_ | 3 | 1.015 (0.977-1.055) |
| PM_10_ | O_3_ | 4 | 1.011 (0.973-1.050) |
| PM_10_ | O_3_ | 5 | 0.995 (0.967-1.024) |
| PM_10_ | O_3_ | 6 | 0.972 (0.939-1.006) |
| PM_10_ | O_3_ | 7 | 0.946 (0.889-1.008) |
| PM_10_ | CO | 0 | 0.966 (0.896-1.042) |
| PM_10_ | CO | 1 | 0.989 (0.949-1.031) |
| PM_10_ | CO | 2 | 1.008 (0.974-1.043) |
| PM_10_ | CO | 3 | 1.018 (0.972-1.065) |
| PM_10_ | CO | 4 | 1.013 (0.968-1.060) |
| PM_10_ | CO | 5 | 0.994 (0.961-1.029) |
| PM_10_ | CO | 6 | 0.967 (0.928-1.007) |
| PM_10_ | CO | 7 | 0.935 (0.868-1.008) |
| SO_2_ | PM_2.5_ | 0 | 1.053 (0.967-1.147) |
| SO_2_ | PM_2.5_ | 1 | 1.038 (0.993-1.086) |
| SO_2_ | PM_2.5_ | 2 | 1.023 (0.983-1.065) |
| SO_2_ | PM_2.5_ | 3 | 1.007 (0.954-1.064) |
| SO_2_ | PM_2.5_ | 4 | 0.991 (0.939-1.046) |
| SO_2_ | PM_2.5_ | 5 | 0.974 (0.935-1.014) |
| SO_2_ | PM_2.5_ | 6 | 0.956 (0.905-1.010) |
| SO_2_ | PM_2.5_ | 7 | 0.938 (0.847-1.039) |
| SO_2_ | PM_10_ | 0 | 1.056 (0.970-1.149) |
| SO_2_ | PM_10_ | 1 | 1.040 (0.994-1.087) |
| SO_2_ | PM_10_ | 2 | 1.024 (0.984-1.065) |
| SO_2_ | PM_10_ | 3 | 1.007 (0.954-1.064) |
| SO_2_ | PM_10_ | 4 | 0.991 (0.939-1.045) |
| SO_2_ | PM_10_ | 5 | 0.974 (0.935-1.014) |
| SO_2_ | PM_10_ | 6 | 0.957 (0.906-1.011) |
| SO_2_ | PM_10_ | 7 | 0.940 (0.849-1.040) |
| SO_2_ | NO_2_ | 0 | 1.034 (0.947-1.128) |
| SO_2_ | NO_2_ | 1 | 1.031 (0.984-1.079) |
| SO_2_ | NO_2_ | 2 | 1.024 (0.983-1.068) |
| SO_2_ | NO_2_ | 3 | 1.013 (0.957-1.071) |
| SO_2_ | NO_2_ | 4 | 0.993 (0.939-1.049) |
| SO_2_ | NO_2_ | 5 | 0.965 (0.925-1.006) |
| SO_2_ | NO_2_ | 6 | 0.933 (0.880-0.989) |
| SO_2_ | NO_2_ | 7 | 0.899 (0.807-1.001) |
| SO_2_ | O_3_ | 0 | 1.051 (0.966-1.142) |
| SO_2_ | O_3_ | 1 | 1.038 (0.994-1.085) |
| SO_2_ | O_3_ | 2 | 1.025 (0.986-1.066) |
| SO_2_ | O_3_ | 3 | 1.010 (0.957-1.066) |
| SO_2_ | O_3_ | 4 | 0.992 (0.941-1.046) |
| SO_2_ | O_3_ | 5 | 0.972 (0.934-1.011) |
| SO_2_ | O_3_ | 6 | 0.949 (0.900-1.002) |
| SO_2_ | O_3_ | 7 | 0.927 (0.839-1.024) |
| SO_2_ | CO | 0 | 1.053 (0.965-1.148) |
| SO_2_ | CO | 1 | 1.040 (0.994-1.088) |
| SO_2_ | CO | 2 | 1.026 (0.985-1.068) |
| SO_2_ | CO | 3 | 1.010 (0.955-1.068) |
| SO_2_ | CO | 4 | 0.991 (0.938-1.047) |
| SO_2_ | CO | 5 | 0.970 (0.931-1.010) |
| SO_2_ | CO | 6 | 0.947 (0.894-1.002) |
| SO_2_ | CO | 7 | 0.923 (0.831-1.026) |
| SO_2_ | PM_2.5_ | 0 | 1.040 (0.967-1.119) |
| SO_2_ | PM_2.5_ | 1 | 1.025 (0.987-1.064) |
| SO_2_ | PM_2.5_ | 2 | 1.012 (0.979-1.047) |
| SO_2_ | PM_2.5_ | 3 | 1.006 (0.960-1.054) |
| SO_2_ | PM_2.5_ | 4 | 1.008 (0.962-1.056) |
| SO_2_ | PM_2.5_ | 5 | 1.018 (0.985-1.053) |
| SO_2_ | PM_2.5_ | 6 | 1.035 (0.995-1.077) |
| SO_2_ | PM_2.5_ | 7 | 1.055 (0.978-1.138) |
| SO_2_ | PM_10_ | 0 | 1.048 (0.974-1.128) |
| SO_2_ | PM_10_ | 1 | 1.030 (0.992-1.069) |
| SO_2_ | PM_10_ | 2 | 1.015 (0.982-1.049) |
| SO_2_ | PM_10_ | 3 | 1.006 (0.961-1.054) |
| SO_2_ | PM_10_ | 4 | 1.008 (0.962-1.055) |
| SO_2_ | PM_10_ | 5 | 1.018 (0.985-1.053) |
| SO_2_ | PM_10_ | 6 | 1.036 (0.996-1.077) |
| SO_2_ | PM_10_ | 7 | 1.057 (0.981-1.140) |
| SO_2_ | SO_2_ | 0 | 1.020 (0.950-1.095) |
| SO_2_ | SO_2_ | 1 | 1.015 (0.978-1.052) |
| SO_2_ | SO_2_ | 2 | 1.011 (0.979-1.044) |
| SO_2_ | SO_2_ | 3 | 1.010 (0.966-1.057) |
| SO_2_ | SO_2_ | 4 | 1.014 (0.969-1.061) |
| SO_2_ | SO_2_ | 5 | 1.022 (0.990-1.056) |
| SO_2_ | SO_2_ | 6 | 1.034 (0.996-1.074) |
| SO_2_ | SO_2_ | 7 | 1.047 (0.973-1.127) |
| SO_2_ | O_3_ | 0 | 1.037 (0.969-1.110) |
| SO_2_ | O_3_ | 1 | 1.027 (0.991-1.063) |
| SO_2_ | O_3_ | 2 | 1.018 (0.987-1.050) |
| SO_2_ | O_3_ | 3 | 1.012 (0.969-1.057) |
| SO_2_ | O_3_ | 4 | 1.009 (0.966-1.054) |
| SO_2_ | O_3_ | 5 | 1.011 (0.980-1.042) |
| SO_2_ | O_3_ | 6 | 1.015 (0.980-1.051) |
| SO_2_ | O_3_ | 7 | 1.020 (0.952-1.094) |
| SO_2_ | CO | 0 | 1.043 (0.967-1.125) |
| SO_2_ | CO | 1 | 1.028 (0.989-1.069) |
| SO_2_ | CO | 2 | 1.016 (0.982-1.052) |
| SO_2_ | CO | 3 | 1.009 (0.961-1.059) |
| SO_2_ | CO | 4 | 1.008 (0.960-1.058) |
| SO_2_ | CO | 5 | 1.014 (0.979-1.049) |
| SO_2_ | CO | 6 | 1.024 (0.983-1.066) |
| SO_2_ | CO | 7 | 1.037 (0.959-1.121) |
| O_3_ | PM_2.5_ | 0 | 0.953 (0.872-1.041) |
| O_3_ | PM_2.5_ | 1 | 0.989 (0.943-1.038) |
| O_3_ | PM_2.5_ | 2 | 1.021 (0.982-1.062) |
| O_3_ | PM_2.5_ | 3 | 1.042 (0.989-1.097) |
| O_3_ | PM_2.5_ | 4 | 1.046 (0.993-1.102) |
| O_3_ | PM_2.5_ | 5 | 1.033 (0.993-1.075) |
| O_3_ | PM_2.5_ | 6 | 1.009 (0.961-1.060) |
| O_3_ | PM_2.5_ | 7 | 0.980 (0.896-1.072) |
| O_3_ | PM_10_ | 0 | 0.955 (0.875-1.042) |
| O_3_ | PM_10_ | 1 | 0.991 (0.945-1.039) |
| O_3_ | PM_10_ | 2 | 1.022 (0.984-1.063) |
| O_3_ | PM_10_ | 3 | 1.043 (0.991-1.098) |
| O_3_ | PM_10_ | 4 | 1.047 (0.995-1.102) |
| O_3_ | PM_10_ | 5 | 1.033 (0.994-1.074) |
| O_3_ | PM_10_ | 6 | 1.008 (0.960-1.058) |
| O_3_ | PM_10_ | 7 | 0.978 (0.895-1.068) |
| O_3_ | NO_2_ | 0 | 0.944 (0.865-1.029) |
| O_3_ | NO_2_ | 1 | 0.985 (0.940-1.032) |
| O_3_ | NO_2_ | 2 | 1.021 (0.983-1.060) |
| O_3_ | NO_2_ | 3 | 1.044 (0.993-1.099) |
| O_3_ | NO_2_ | 4 | 1.047 (0.996-1.102) |
| O_3_ | NO_2_ | 5 | 1.030 (0.992-1.071) |
| O_3_ | NO_2_ | 6 | 1.000 (0.954-1.049) |
| O_3_ | NO_2_ | 7 | 0.965 (0.884-1.053) |
| O_3_ | SO_2_ | 0 | 0.948 (0.870-1.033) |
| O_3_ | SO_2_ | 1 | 0.988 (0.943-1.035) |
| O_3_ | SO_2_ | 2 | 1.023 (0.985-1.062) |
| O_3_ | SO_2_ | 3 | 1.046 (0.994-1.100) |
| O_3_ | SO_2_ | 4 | 1.049 (0.997-1.103) |
| O_3_ | SO_2_ | 5 | 1.033 (0.994-1.073) |
| O_3_ | SO_2_ | 6 | 1.004 (0.957-1.052) |
| O_3_ | SO_2_ | 7 | 0.969 (0.888-1.058) |
| O_3_ | CO | 0 | 0.952 (0.872-1.039) |
| O_3_ | CO | 1 | 0.989 (0.943-1.037) |
| O_3_ | CO | 2 | 1.022 (0.983-1.062) |
| O_3_ | CO | 3 | 1.043 (0.990-1.098) |
| O_3_ | CO | 4 | 1.045 (0.993-1.100) |
| O_3_ | CO | 5 | 1.029 (0.990-1.070) |
| O_3_ | CO | 6 | 1.002 (0.954-1.052) |
| O_3_ | CO | 7 | 0.969 (0.887-1.059) |
| CO | PM_2.5_ | 0 | 1.000 (0.912-1.098) |
| CO | PM_2.5_ | 1 | 1.000 (0.951-1.052) |
| CO | PM_2.5_ | 2 | 1.000 (0.960-1.042) |
| CO | PM_2.5_ | 3 | 1.003 (0.949-1.059) |
| CO | PM_2.5_ | 4 | 1.008 (0.954-1.064) |
| CO | PM_2.5_ | 5 | 1.016 (0.976-1.057) |
| CO | PM_2.5_ | 6 | 1.026 (0.977-1.077) |
| CO | PM_2.5_ | 7 | 1.037 (0.946-1.137) |
| CO | PM_10_ | 0 | 1.007 (0.928-1.094) |
| CO | PM_10_ | 1 | 1.006 (0.962-1.052) |
| CO | PM_10_ | 2 | 1.005 (0.968-1.043) |
| CO | PM_10_ | 3 | 1.005 (0.957-1.056) |
| CO | PM_10_ | 4 | 1.007 (0.959-1.058) |
| CO | PM_10_ | 5 | 1.011 (0.976-1.048) |
| CO | PM_10_ | 6 | 1.016 (0.973-1.061) |
| CO | PM_10_ | 7 | 1.022 (0.942-1.108) |
| CO | NO_2_ | 0 | 0.968 (0.896-1.045) |
| CO | NO_2_ | 1 | 0.986 (0.945-1.028) |
| CO | NO_2_ | 2 | 1.001 (0.967-1.036) |
| CO | NO_2_ | 3 | 1.010 (0.965-1.057) |
| CO | NO_2_ | 4 | 1.010 (0.965-1.057) |
| CO | NO_2_ | 5 | 1.000 (0.967-1.035) |
| CO | NO_2_ | 6 | 0.985 (0.945-1.027) |
| CO | NO_2_ | 7 | 0.967 (0.895-1.044) |
| CO | SO_2_ | 0 | 0.979 (0.912-1.052) |
| CO | SO_2_ | 1 | 0.994 (0.956-1.034) |
| CO | SO_2_ | 2 | 1.007 (0.976-1.039) |
| CO | SO_2_ | 3 | 1.016 (0.974-1.059) |
| CO | SO_2_ | 4 | 1.019 (0.977-1.062) |
| CO | SO_2_ | 5 | 1.015 (0.984-1.047) |
| CO | SO_2_ | 6 | 1.007 (0.970-1.046) |
| CO | SO_2_ | 7 | 0.997 (0.929-1.070) |
| CO | O_3_ | 0 | 0.997 (0.930-1.070) |
| CO | O_3_ | 1 | 1.003 (0.965-1.042) |
| CO | O_3_ | 2 | 1.007 (0.976-1.039) |
| CO | O_3_ | 3 | 1.009 (0.968-1.052) |
| CO | O_3_ | 4 | 1.007 (0.967-1.050) |
| CO | O_3_ | 5 | 1.002 (0.972-1.033) |
| CO | O_3_ | 6 | 0.994 (0.959-1.032) |
| CO | O_3_ | 7 | 0.986 (0.920-1.056) |

Definition of abbreviations: CO, carbon monoxide; CIs, confidence intervals; DR-TB, Drug resistant tuberculosis; IQR, interquartile range; NO_2_, nitrogen dioxide; O_3_,ozone; PM_2.5_, particulate matter with an aerodynamic diameter <2.5 mm; PM_10_, particulate matter with an aerodynamic diameter <10 mm; RR, Relative risks; SO_2_, sulfur dioxide.

This model was adjusted for daily relative humidity, average temperature, and holidays.

Table S3 Cumulative RR (95% CIs) for the association between first-time outpatient visits for acute exacerbations of DR-TB and air pollutants concentrations with each IQR increase based on two-pollutants models.

| Air pollutants | Adjusted pollutants | Lag day | RR(95%CI) |
| --- | --- | --- | --- |
| PM_2.5_ | PM_10_ | 01 | 1.060 (0.865-1.300) |
| PM_2.5_ | PM_10_ | 02 | 1.079 (0.859-1.355) |
| PM_2.5_ | PM_10_ | 03 | 1.090 (0.855-1.389) |
| PM_2.5_ | PM_10_ | 04 | 1.094 (0.836-1.432) |
| PM_2.5_ | PM_10_ | 05 | 1.093 (0.816-1.464) |
| PM_2.5_ | PM_10_ | 06 | 1.087 (0.802-1.471) |
| PM_2.5_ | PM_10_ | 07 | 1.076 (0.772-1.499) |
| PM_2.5_ | NO_2_ | 01 | 0.951 (0.851-1.062) |
| PM_2.5_ | NO_2_ | 02 | 0.955 (0.844-1.082) |
| PM_2.5_ | NO_2_ | 03 | 0.969 (0.850-1.105) |
| PM_2.5_ | NO_2_ | 04 | 0.978 (0.847-1.128) |
| PM_2.5_ | NO_2_ | 05 | 0.967 (0.829-1.128) |
| PM_2.5_ | NO_2_ | 06 | 0.929 (0.792-1.089) |
| PM_2.5_ | NO_2_ | 07 | 0.862 (0.722-1.029) |
| PM_2.5_ | SO_2_ | 01 | 0.967 (0.871-1.073) |
| PM_2.5_ | SO_2_ | 02 | 0.978 (0.870-1.100) |
| PM_2.5_ | SO_2_ | 03 | 1.000 (0.884-1.131) |
| PM_2.5_ | SO_2_ | 04 | 1.019 (0.892-1.165) |
| PM_2.5_ | SO_2_ | 05 | 1.024 (0.887-1.181) |
| PM_2.5_ | SO_2_ | 06 | 1.005 (0.867-1.165) |
| PM_2.5_ | SO_2_ | 07 | 0.961 (0.815-1.132) |
| PM_2.5_ | O_3_ | 01 | 0.989 (0.891-1.098) |
| PM_2.5_ | O_3_ | 02 | 0.999 (0.888-1.124) |
| PM_2.5_ | O_3_ | 03 | 1.013 (0.894-1.147) |
| PM_2.5_ | O_3_ | 04 | 1.020 (0.891-1.168) |
| PM_2.5_ | O_3_ | 05 | 1.013 (0.876-1.171) |
| PM_2.5_ | O_3_ | 06 | 0.985 (0.848-1.144) |
| PM_2.5_ | O_3_ | 07 | 0.934 (0.792-1.102) |
| PM_2.5_ | CO | 01 | 0.980 (0.854-1.124) |
| PM_2.5_ | CO | 02 | 0.994 (0.853-1.160) |
| PM_2.5_ | CO | 03 | 1.015 (0.863-1.192) |
| PM_2.5_ | CO | 04 | 1.026 (0.862-1.223) |
| PM_2.5_ | CO | 05 | 1.015 (0.842-1.224) |
| PM_2.5_ | CO | 06 | 0.972 (0.803-1.177) |
| PM_2.5_ | CO | 07 | 0.897 (0.726-1.109) |
| PM_10_ | PM_2.5_ | 01 | 0.919 (0.760-1.112) |
| PM_10_ | PM_2.5_ | 02 | 0.917 (0.742-1.133) |
| PM_10_ | PM_2.5_ | 03 | 0.928 (0.741-1.162) |
| PM_10_ | PM_2.5_ | 04 | 0.940 (0.731-1.208) |
| PM_10_ | PM_2.5_ | 05 | 0.941 (0.714-1.240) |
| PM_10_ | PM_2.5_ | 06 | 0.922 (0.691-1.229) |
| PM_10_ | PM_2.5_ | 07 | 0.880 (0.642-1.205) |
| PM_10_ | NO_2_ | 01 | 0.927 (0.836-1.029) |
| PM_10_ | NO_2_ | 02 | 0.928 (0.827-1.042) |
| PM_10_ | NO_2_ | 03 | 0.941 (0.833-1.064) |
| PM_10_ | NO_2_ | 04 | 0.951 (0.832-1.088) |
| PM_10_ | NO_2_ | 05 | 0.942 (0.815-1.090) |
| PM_10_ | NO_2_ | 06 | 0.905 (0.777-1.054) |
| PM_10_ | NO_2_ | 07 | 0.839 (0.707-0.995) |
| PM_10_ | SO_2_ | 01 | 0.951 (0.864-1.048) |
| PM_10_ | SO_2_ | 02 | 0.960 (0.862-1.069) |
| PM_10_ | SO_2_ | 03 | 0.980 (0.875-1.097) |
| PM_10_ | SO_2_ | 04 | 0.999 (0.883-1.130) |
| PM_10_ | SO_2_ | 05 | 1.003 (0.878-1.147) |
| PM_10_ | SO_2_ | 06 | 0.984 (0.856-1.130) |
| PM_10_ | SO_2_ | 07 | 0.937 (0.803-1.094) |
| PM_10_ | O_3_ | 01 | 0.969 (0.881-1.067) |
| PM_10_ | O_3_ | 02 | 0.977 (0.878-1.088) |
| PM_10_ | O_3_ | 03 | 0.992 (0.886-1.111) |
| PM_10_ | O_3_ | 04 | 1.002 (0.885-1.135) |
| PM_10_ | O_3_ | 05 | 0.997 (0.872-1.141) |
| PM_10_ | O_3_ | 06 | 0.970 (0.843-1.115) |
| PM_10_ | O_3_ | 07 | 0.918 (0.786-1.071) |
| PM_10_ | CO | 01 | 0.956 (0.853-1.070) |
| PM_10_ | CO | 02 | 0.963 (0.849-1.092) |
| PM_10_ | CO | 03 | 0.980 (0.858-1.119) |
| PM_10_ | CO | 04 | 0.993 (0.858-1.149) |
| PM_10_ | CO | 05 | 0.987 (0.843-1.156) |
| PM_10_ | CO | 06 | 0.954 (0.810-1.123) |
| PM_10_ | CO | 07 | 0.893 (0.745-1.069) |
| SO_2_ | PM_2.5_ | 01 | 1.094 (0.964-1.241) |
| SO_2_ | PM_2.5_ | 02 | 1.119 (0.975-1.284) |
| SO_2_ | PM_2.5_ | 03 | 1.127 (0.975-1.303) |
| SO_2_ | PM_2.5_ | 04 | 1.117 (0.952-1.311) |
| SO_2_ | PM_2.5_ | 05 | 1.088 (0.915-1.292) |
| SO_2_ | PM_2.5_ | 06 | 1.040 (0.871-1.241) |
| SO_2_ | PM_2.5_ | 07 | 0.975 (0.794-1.198) |
| SO_2_ | PM_10_ | 01 | 1.098 (0.968-1.244) |
| SO_2_ | PM_10_ | 02 | 1.124 (0.980-1.289) |
| SO_2_ | PM_10_ | 03 | 1.132 (0.981-1.307) |
| SO_2_ | PM_10_ | 04 | 1.122 (0.957-1.315) |
| SO_2_ | PM_10_ | 05 | 1.092 (0.920-1.297) |
| SO_2_ | PM_10_ | 06 | 1.045 (0.876-1.246) |
| SO_2_ | PM_10_ | 07 | 0.982 (0.801-1.205) |
| SO_2_ | NO_2_ | 01 | 1.065 (0.937-1.212) |
| SO_2_ | NO_2_ | 02 | 1.091 (0.948-1.256) |
| SO_2_ | NO_2_ | 03 | 1.105 (0.953-1.281) |
| SO_2_ | NO_2_ | 04 | 1.097 (0.931-1.292) |
| SO_2_ | NO_2_ | 05 | 1.058 (0.887-1.262) |
| SO_2_ | NO_2_ | 06 | 0.987 (0.824-1.182) |
| SO_2_ | NO_2_ | 07 | 0.887 (0.718-1.096) |
| SO_2_ | O_3_ | 01 | 1.091 (0.964-1.234) |
| SO_2_ | O_3_ | 02 | 1.118 (0.977-1.280) |
| SO_2_ | O_3_ | 03 | 1.130 (0.980-1.302) |
| SO_2_ | O_3_ | 04 | 1.121 (0.958-1.311) |
| SO_2_ | O_3_ | 05 | 1.089 (0.920-1.289) |
| SO_2_ | O_3_ | 06 | 1.034 (0.870-1.229) |
| SO_2_ | O_3_ | 07 | 0.958 (0.784-1.170) |
| SO_2_ | CO | 01 | 1.094 (0.963-1.244) |
| SO_2_ | CO | 02 | 1.123 (0.977-1.290) |
| SO_2_ | CO | 03 | 1.133 (0.980-1.311) |
| SO_2_ | CO | 04 | 1.123 (0.956-1.320) |
| SO_2_ | CO | 05 | 1.089 (0.916-1.295) |
| SO_2_ | CO | 06 | 1.031 (0.863-1.231) |
| SO_2_ | CO | 07 | 0.952 (0.774-1.171) |
| NO_2_ | PM_2.5_ | 01 | 1.066 (0.957-1.187) |
| NO_2_ | PM_2.5_ | 02 | 1.079 (0.961-1.212) |
| NO_2_ | PM_2.5_ | 03 | 1.085 (0.962-1.224) |
| NO_2_ | PM_2.5_ | 04 | 1.094 (0.958-1.249) |
| NO_2_ | PM_2.5_ | 05 | 1.114 (0.966-1.285) |
| NO_2_ | PM_2.5_ | 06 | 1.153 (1.000-1.329) |
| NO_2_ | PM_2.5_ | 07 | 1.216 (1.045-1.416) |
| NO_2_ | PM_10_ | 01 | 1.079 (0.969-1.202) |
| NO_2_ | PM_10_ | 02 | 1.095 (0.975-1.230) |
| NO_2_ | PM_10_ | 03 | 1.102 (0.977-1.243) |
| NO_2_ | PM_10_ | 04 | 1.110 (0.972-1.268) |
| NO_2_ | PM_10_ | 05 | 1.131 (0.980-1.304) |
| NO_2_ | PM_10_ | 06 | 1.171 (1.015-1.351) |
| NO_2_ | PM_10_ | 07 | 1.238 (1.063-1.443) |
| NO_2_ | SO_2_ | 01 | 1.035 (0.933-1.149) |
| NO_2_ | SO_2_ | 02 | 1.046 (0.935-1.171) |
| NO_2_ | SO_2_ | 03 | 1.057 (0.942-1.187) |
| NO_2_ | SO_2_ | 04 | 1.072 (0.944-1.218) |
| NO_2_ | SO_2_ | 05 | 1.096 (0.956-1.257) |
| NO_2_ | SO_2_ | 06 | 1.134 (0.990-1.298) |
| NO_2_ | SO_2_ | 07 | 1.187 (1.028-1.371) |
| NO_2_ | O_3_ | 01 | 1.065 (0.963-1.177) |
| NO_2_ | O_3_ | 02 | 1.084 (0.973-1.207) |
| NO_2_ | O_3_ | 03 | 1.096 (0.981-1.225) |
| NO_2_ | O_3_ | 04 | 1.106 (0.979-1.250) |
| NO_2_ | O_3_ | 05 | 1.118 (0.981-1.276) |
| NO_2_ | O_3_ | 06 | 1.135 (0.996-1.293) |
| NO_2_ | O_3_ | 07 | 1.158 (1.009-1.329) |
| NO_2_ | CO | 01 | 1.073 (0.959-1.199) |
| NO_2_ | CO | 02 | 1.090 (0.967-1.230) |
| NO_2_ | CO | 03 | 1.100 (0.971-1.246) |
| NO_2_ | CO | 04 | 1.109 (0.967-1.271) |
| NO_2_ | CO | 05 | 1.124 (0.970-1.302) |
| NO_2_ | CO | 06 | 1.151 (0.994-1.332) |
| NO_2_ | CO | 07 | 1.193 (1.022-1.394) |
| O_3_ | PM_2.5_ | 01 | 0.943 (0.825-1.077) |
| O_3_ | PM_2.5_ | 02 | 0.962 (0.830-1.116) |
| O_3_ | PM_2.5_ | 03 | 1.003 (0.859-1.171) |
| O_3_ | PM_2.5_ | 04 | 1.049 (0.887-1.240) |
| O_3_ | PM_2.5_ | 05 | 1.084 (0.906-1.298) |
| O_3_ | PM_2.5_ | 06 | 1.094 (0.908-1.318) |
| O_3_ | PM_2.5_ | 07 | 1.073 (0.870-1.322) |
| O_3_ | PM_10_ | 01 | 0.946 (0.829-1.079) |
| O_3_ | PM_10_ | 02 | 0.967 (0.835-1.119) |
| O_3_ | PM_10_ | 03 | 1.009 (0.866-1.175) |
| O_3_ | PM_10_ | 04 | 1.056 (0.895-1.245) |
| O_3_ | PM_10_ | 05 | 1.091 (0.914-1.302) |
| O_3_ | PM_10_ | 06 | 1.099 (0.915-1.321) |
| O_3_ | PM_10_ | 07 | 1.075 (0.874-1.322) |
| O_3_ | NO_2_ | 01 | 0.930 (0.816-1.059) |
| O_3_ | NO_2_ | 02 | 0.949 (0.821-1.096) |
| O_3_ | NO_2_ | 03 | 0.991 (0.852-1.152) |
| O_3_ | NO_2_ | 04 | 1.038 (0.882-1.221) |
| O_3_ | NO_2_ | 05 | 1.069 (0.898-1.273) |
| O_3_ | NO_2_ | 06 | 1.070 (0.893-1.281) |
| O_3_ | NO_2_ | 07 | 1.032 (0.842-1.265) |
| O_3_ | SO_2_ | 01 | 0.937 (0.823-1.066) |
| O_3_ | SO_2_ | 02 | 0.958 (0.830-1.106) |
| O_3_ | SO_2_ | 03 | 1.002 (0.862-1.164) |
| O_3_ | SO_2_ | 04 | 1.051 (0.894-1.236) |
| O_3_ | SO_2_ | 05 | 1.085 (0.912-1.291) |
| O_3_ | SO_2_ | 06 | 1.089 (0.910-1.304) |
| O_3_ | SO_2_ | 07 | 1.055 (0.861-1.293) |
| O_3_ | CO | 01 | 0.941 (0.825-1.074) |
| O_3_ | CO | 02 | 0.961 (0.830-1.113) |
| O_3_ | CO | 03 | 1.002 (0.860-1.168) |
| O_3_ | CO | 04 | 1.048 (0.888-1.236) |
| O_3_ | CO | 05 | 1.079 (0.903-1.288) |
| O_3_ | CO | 06 | 1.080 (0.899-1.299) |
| O_3_ | CO | 07 | 1.047 (0.851-1.289) |
| CO | PM_2.5_ | 01 | 1.000 (0.870-1.150) |
| CO | PM_2.5_ | 02 | 1.001 (0.857-1.169) |
| CO | PM_2.5_ | 03 | 1.003 (0.853-1.180) |
| CO | PM_2.5_ | 04 | 1.011 (0.848-1.205) |
| CO | PM_2.5_ | 05 | 1.027 (0.852-1.238) |
| CO | PM_2.5_ | 06 | 1.053 (0.872-1.273) |
| CO | PM_2.5_ | 07 | 1.092 (0.888-1.344) |
| CO | PM_10_ | 01 | 1.014 (0.896-1.147) |
| CO | PM_10_ | 02 | 1.019 (0.888-1.169) |
| CO | PM_10_ | 03 | 1.024 (0.886-1.184) |
| CO | PM_10_ | 04 | 1.032 (0.881-1.208) |
| CO | PM_10_ | 05 | 1.043 (0.882-1.234) |
| CO | PM_10_ | 06 | 1.060 (0.893-1.258) |
| CO | PM_10_ | 07 | 1.083 (0.899-1.304) |
| CO | NO_2_ | 01 | 0.954 (0.850-1.072) |
| CO | NO_2_ | 02 | 0.955 (0.839-1.087) |
| CO | NO_2_ | 03 | 0.965 (0.842-1.105) |
| CO | NO_2_ | 04 | 0.974 (0.840-1.129) |
| CO | NO_2_ | 05 | 0.974 (0.832-1.141) |
| CO | NO_2_ | 06 | 0.960 (0.817-1.127) |
| CO | NO_2_ | 07 | 0.928 (0.777-1.107) |
| CO | SO_2_ | 01 | 0.974 (0.875-1.084) |
| CO | SO_2_ | 02 | 0.981 (0.871-1.105) |
| CO | SO_2_ | 03 | 0.997 (0.880-1.129) |
| CO | SO_2_ | 04 | 1.015 (0.887-1.161) |
| CO | SO_2_ | 05 | 1.030 (0.892-1.190) |
| CO | SO_2_ | 06 | 1.038 (0.896-1.202) |
| CO | SO_2_ | 07 | 1.035 (0.880-1.217) |
| CO | O_3_ | 01 | 1.000 (0.900-1.111) |
| CO | O_3_ | 02 | 1.007 (0.896-1.133) |
| CO | O_3_ | 03 | 1.017 (0.899-1.150) |
| CO | O_3_ | 04 | 1.024 (0.896-1.171) |
| CO | O_3_ | 05 | 1.026 (0.889-1.184) |
| CO | O_3_ | 06 | 1.021 (0.882-1.181) |
| CO | O_3_ | 07 | 1.006 (0.857-1.180) |

Definition of abbreviations: CO, carbon monoxide; CIs, confidence intervals; DR-TB, Drug resistant tuberculosis; IQR, interquartile range; NO_2_, nitrogen dioxide; O_3_,ozone; PM_2.5_, particulate matter with an aerodynamic diameter <2.5 mm; PM_10_, particulate matter with an aerodynamic diameter <10 mm; RR, Relative risks; SO_2_, sulfur dioxide.This model was adjusted for daily relative humidity, average temperature, and holidays.


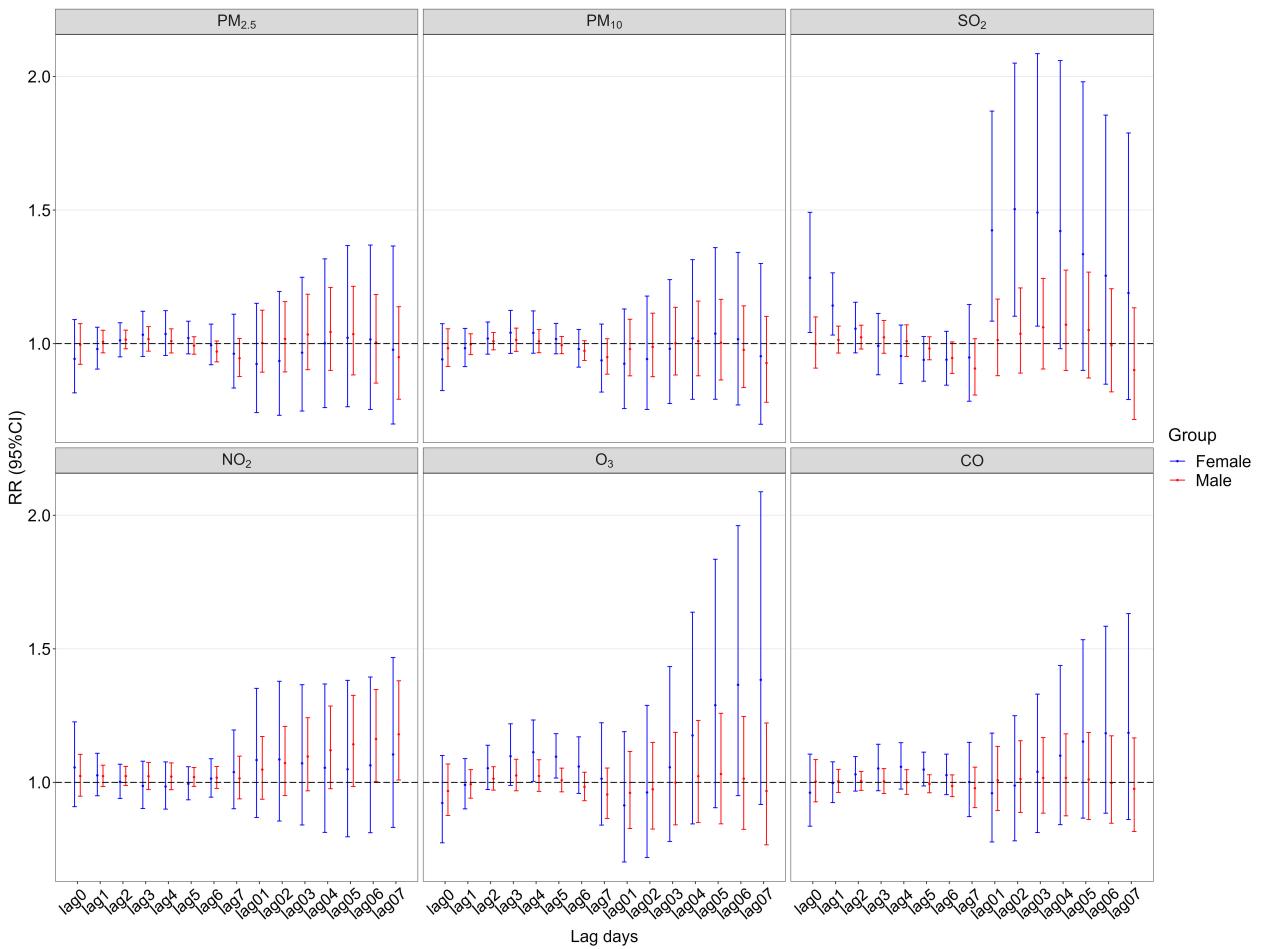


Figure S1. RR (95% CIs) for the association between first-time outpatient visits for acute exacerbations of DR-TB and air pollutants concentrations with each IQR increase based on single-pollutant models stratified by gender

This model was adjusted for daily relative humidity, average temperature, and holidays.


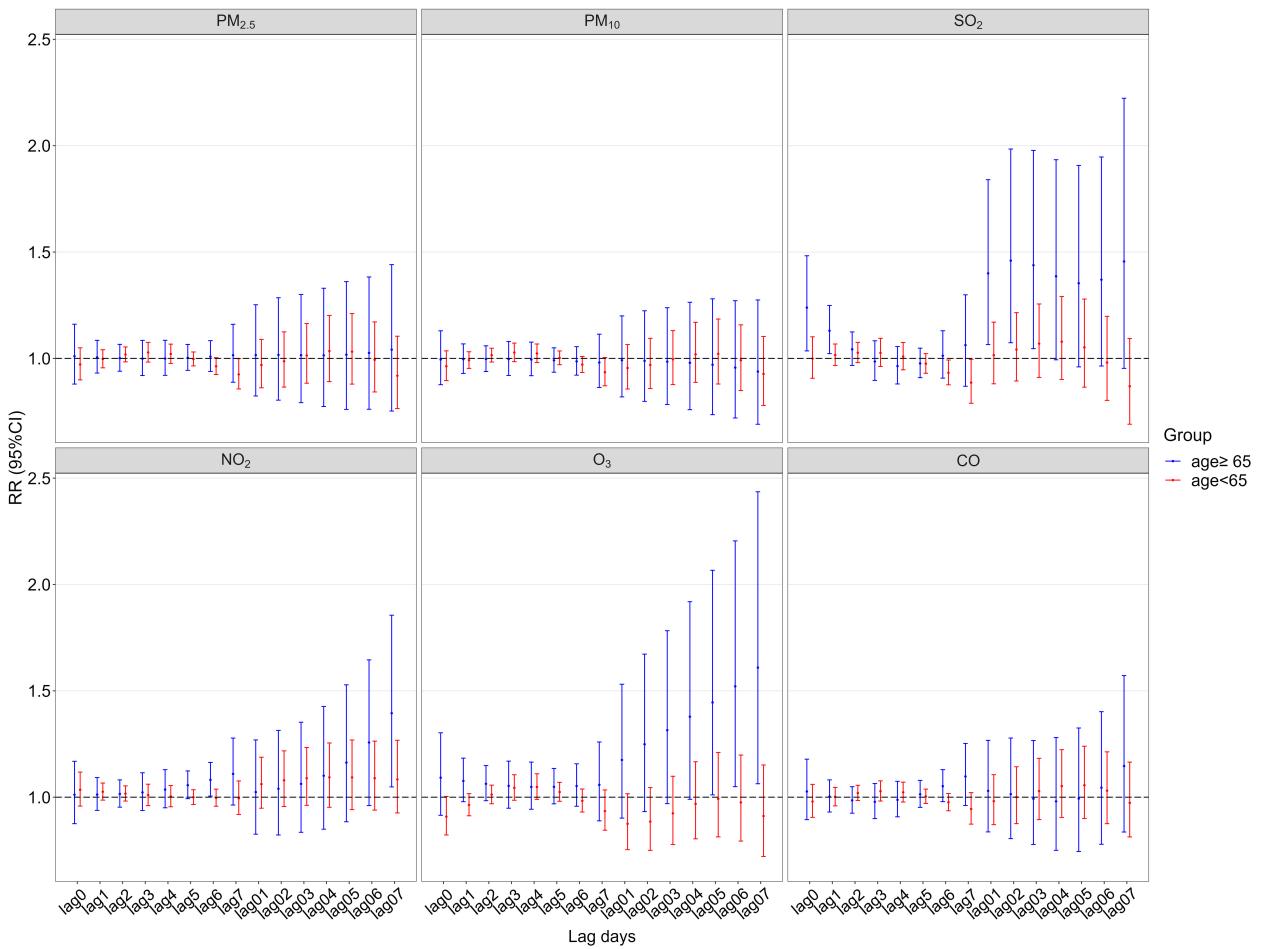


Figure S2. RR (95% CIs) for the association between first-time outpatient visits for acute exacerbations of DR-TB and air pollutants concentrations with each IQR increase based on single-pollutant models stratified by age

This model was adjusted for daily relative humidity, average temperature, and holidays.


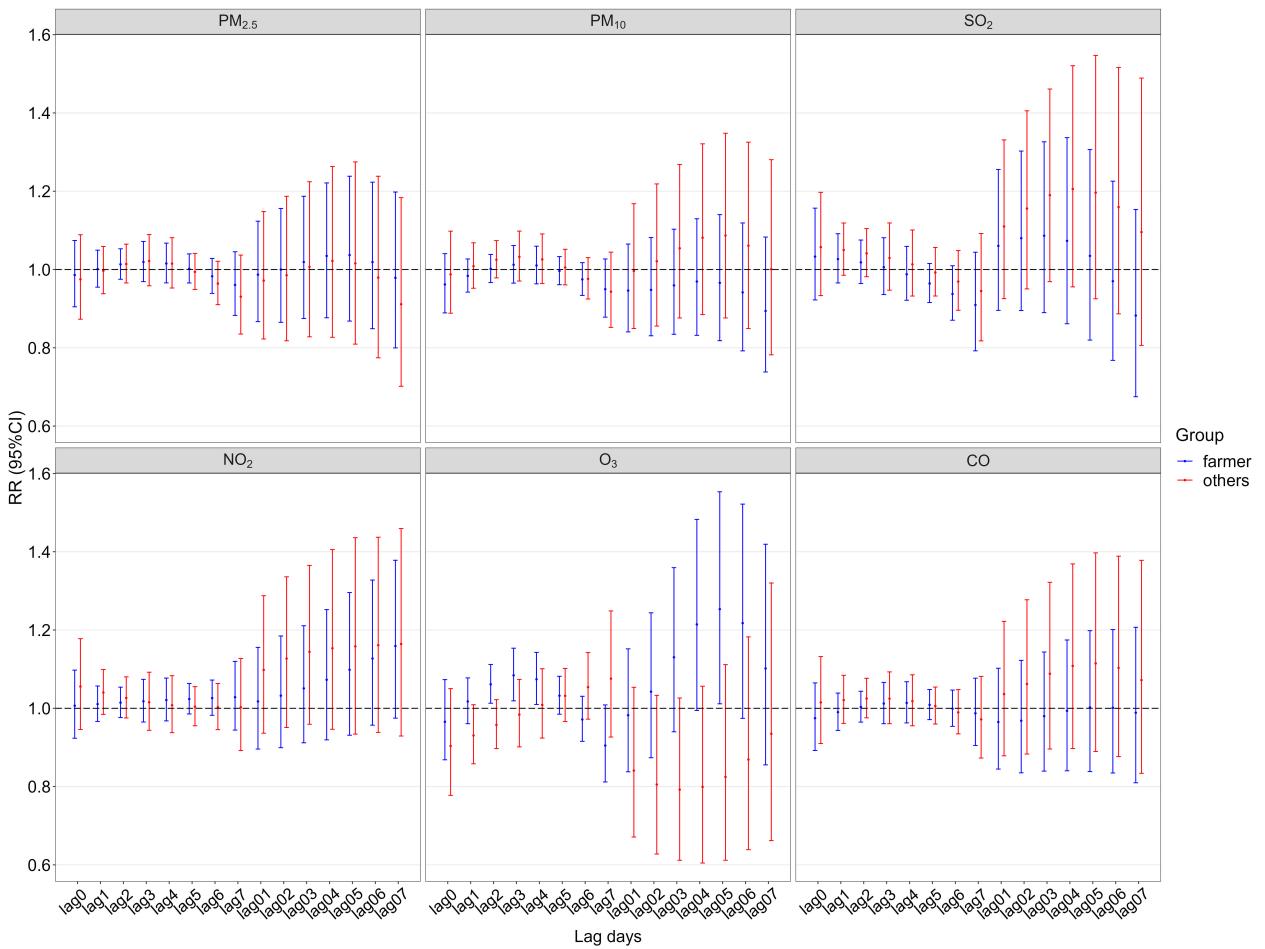


Figure S3. RR (95% CIs) for the association between first-time outpatient visits for acute exacerbations of DR-TB and air pollutants concentrations with each IQR increase based on single-pollutant models stratified by occupation

This model was adjusted for daily relative humidity, average temperature, and holidays.


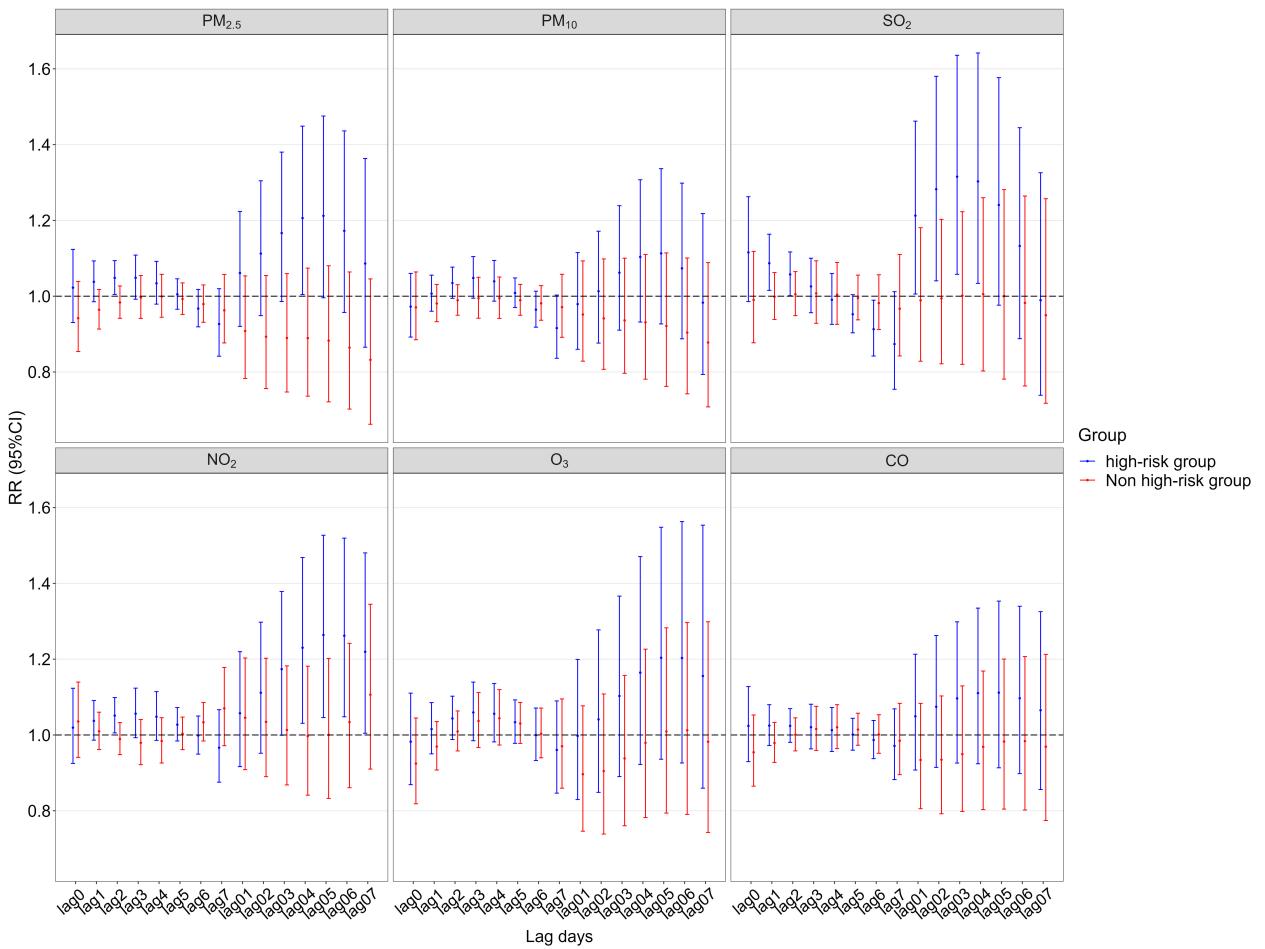


Figure S4. RR (95% CIs) for the association between first-time outpatient visits for DR-TB and air pollutants concentrations with each IQR increase based on single-pollutant models stratified by high-risk subgroup


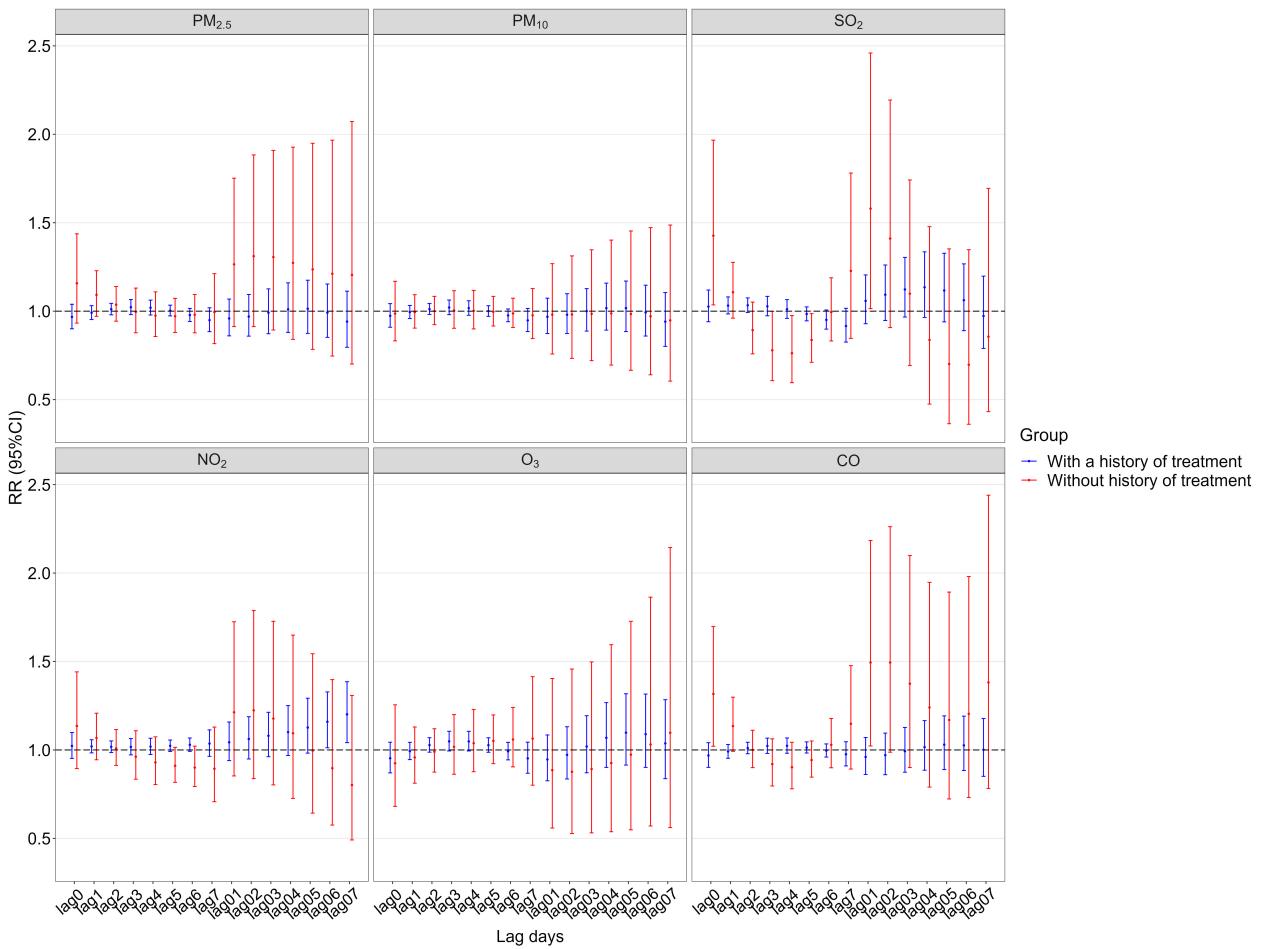


Figure S5. RR (95% CIs) for the association between first-time outpatient visits for DR-TB and air pollutants concentrations with each IQR increase based on single-pollutant models stratified by history of treatment


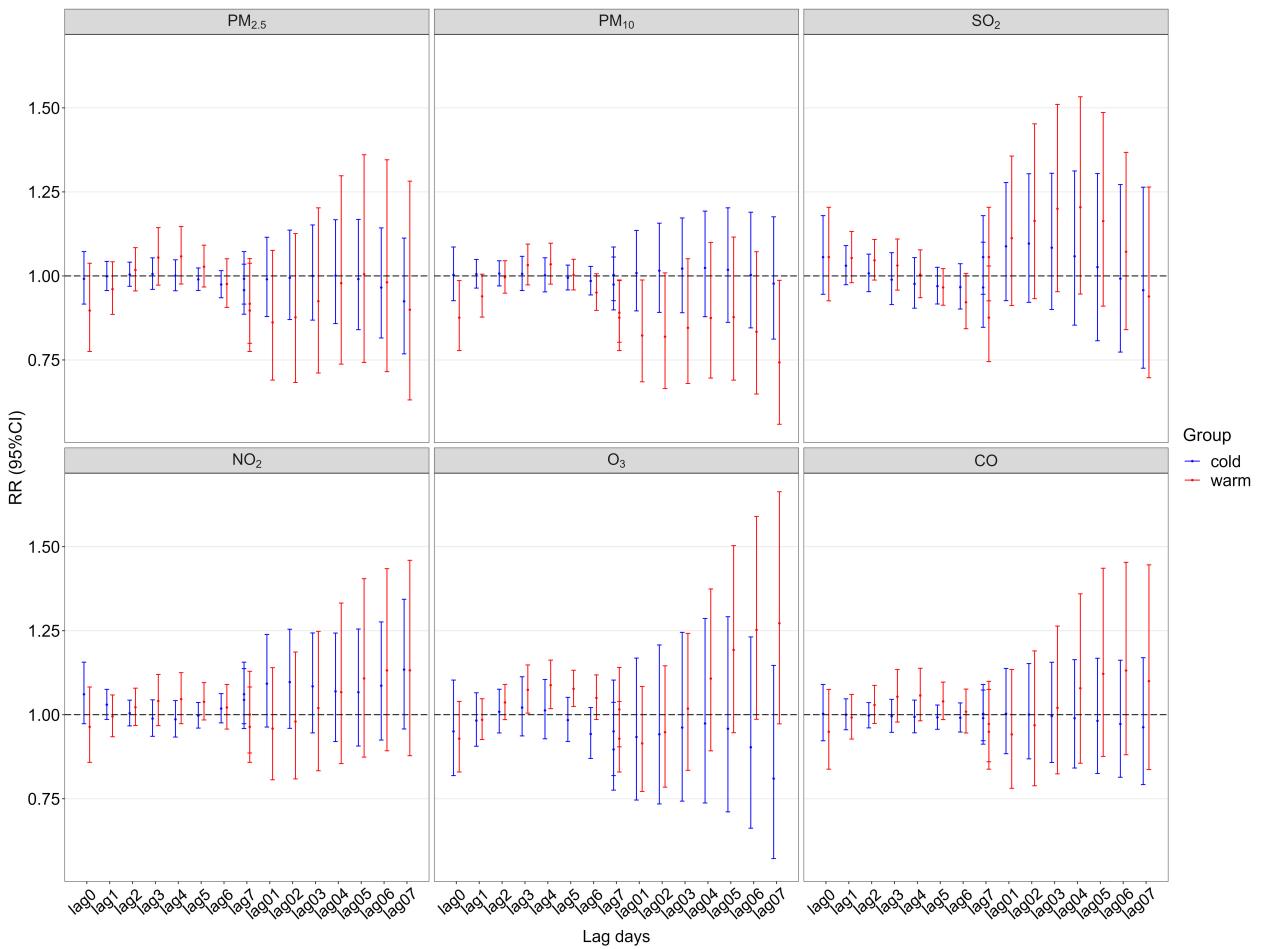


Figure S6. RR (95% CIs) for the association between first-time outpatient visits for DR-TB and air pollutants concentrations with each IQR increase based on single-pollutant models stratified by season
